# Market Forces and Technological Substitutes Cause Fluctuations in the Value of Bat Pest-Control Services for Cotton

**DOI:** 10.1371/journal.pone.0087912

**Published:** 2014-02-03

**Authors:** Laura López-Hoffman, Ruscena Wiederholt, Chris Sansone, Kenneth J. Bagstad, Paul Cryan, Jay E. Diffendorfer, Joshua Goldstein, Kelsie LaSharr, John Loomis, Gary McCracken, Rodrigo A. Medellín, Amy Russell, Darius Semmens

**Affiliations:** 1 School of Natural Resources & the Environment, The University of Arizona, Tucson, Arizona, United States of America; 2 Udall Center for Studies in Public Policy, The University of Arizona, Tucson, Arizona, United States of America; 3 Bayer CropScience, Research Triangle Park, North Carolina, United States of America; 4 United States Geological Survey, Geosciences and Environmental Change Science Center, Denver, Colorado, United States of America; 5 United States Geological Survey, Fort Collins Science Center, Fort Collins, Colorado, United States of America; 6 Department of Human Dimensions of Natural Resources, Colorado State University, Fort Collins, Colorado, United States of America; 7 Department of Agricultural and Resource Economics, Colorado State University, Fort Collins, Colorado, United States of America; 8 Department of Ecology and Evolutionary Biology, University of Tennessee, Knoxville, Tennessee, United States of America; 9 Instituto de Ecología, Universidad Nacional Autónoma de México, Distrito Federal, México; 10 Department of Biology, Grand Valley State University, Allendale, Michigan, United States of America; USDA-Agricultural Research Service, United States of America

## Abstract

Critics of the market-based, ecosystem services approach to biodiversity conservation worry that volatile market conditions and technological substitutes will diminish the value of ecosystem services and obviate the “economic benefits” arguments for conservation. To explore the effects of market forces and substitutes on service values, we assessed how the value of the pest-control services provided by Mexican free-tailed bats (*Tadarida brasiliensis mexicana*) to cotton production in the southwestern U.S. has changed over time. We calculated service values each year from 1990 through 2008 by estimating the value of avoided crop damage and the reduced social and private costs of insecticide use in the presence of bats. Over this period, the ecosystem service value declined by 79% ($19.09 million U.S. dollars) due to the introduction and widespread adoption of Bt (*Bacillus thuringiensis*) cotton transgenically modified to express its own pesticide, falling global cotton prices and the reduction in the number of hectares in the U.S. planted with cotton. Our results demonstrate that fluctuations in market conditions can cause temporal variation in ecosystem service values even when ecosystem function – in this case bat population numbers – is held constant. Evidence is accumulating, however, of the evolution of pest resistance to Bt cotton, suggesting that the value of bat pest-control services may increase again. This gives rise to an economic option value argument for conserving Mexican free-tailed bat populations. We anticipate that these results will spur discussion about the role of ecosystem services in biodiversity conservation in general, and bat conservation in particular.

## Introduction

The underlying goal of market-based, Payments for Ecosystem Services (PES) approaches to conservation is the creation of monetary incentives for the protection of critical ecological processes such as watershed functioning, pollination and natural pest control [1], [2]. Within the conservation community, criticisms about market-based programs range from the ideological – e.g. unease that the approach diminishes nature's intrinsic value [3] – to apprehensions about the nature of the market [3], [4]. The latter criticism stems from the worry there will be no reason to protect ecosystems when their services are no longer perceived to be valuable [3], [4]. Two issues in particular – volatile market conditions and technological substitutes – are the main source of concern about the compatibility of market-based approaches and biodiversity conservation [4]–[6].

The first of these concerns is based on the economic principle that as the supply and demand curves for a market good change, the price of that good also changes. It follows that as the price of a market good fluctuates, the value of its ecosystem service inputs also will vary since ecosystem service values are derived from the demands of users of the services, in this case, cotton producers [7]. McCauley [6] illustrated this concern with the anecdote of a Costa Rican coffee plantation that was converted to pineapple production following world-wide declines in coffee prices. The monetary value to coffee production of the pollinators in the surrounding forest fragments previously had been estimated to be $60,000 USD per year [8]. Because pineapples are propagated and not pollinated, as the need for pollination services disappeared, McCauley [6] worried that the rationale for protecting the forest fragments might have disappeared as well.

The second concern about market-based approaches to conservation arises when manufactured capital is substituted for natural capital [9]. The story of the Zapp potato-chip factory in Louisiana (U.S.A.) illustrates this point. The factory once used a nearby wetland to filter its waste. But, as the potato chip business boomed, and as the volume of waste increased, the cost of using the wetland also increased and the company switched to technological forms of wastewater treatment [9].

In our study – using as an example the pest-control services provided by Mexican free-tailed bats (*Tadarida brasiliensis mexicana*) to cotton production in the U.S. – we demonstrate how bat pest-control values have changed in response to both changing market conditions for cotton and the adoption of a technological substitute for the service. To our knowledge, we present one of the first empirical, time-series analyses of the effects of changing market conditions and of the adoption of technological substitutes on the value of an ecosystem service.

As two-thirds of the more than 1,200 extant bat species are insectivorous [10], bats can provide significant complementary pest-control services, particularly by preying on pests early in the growing season before insecticide use has begun and preventing pest outbreaks [11]. Two studies have estimated the monetary value of the pest-control services of Mexican free-tailed bats in reducing crop damage and lowering the costs of insecticide use in cotton [12], [13]. Using cotton price and acreage data from the mid-2000s, Cleveland et al. [12] estimated an annual pest-control value in an eight-county region of south-central Texas of $121,000 to $1,725,000 USD. Researchers have subsequently applied the estimates from the above studies to different regions in North America [14], [15]. However, no previous study has considered how the value of bat ecosystem services has changed over time in response to shifts in commodity markets or advances in agricultural technology.

Over the last two decades, cotton prices and hectares planted in cotton in the U.S. have declined. The declines are generally attributed to global market forces – including trade barriers falling in the 1990s and increased production of cotton in developing countries [16]. In addition, in 1996, U.S. cotton growers started using Bt (*Bacillus thuringiensis*) cotton, which is transgenically modified to produce proteins that are toxic to susceptible insects [17]. As of 2012, 77% of all cotton grown in the U.S. was Bt-modified (www.ers.usda.gov/Data/BiotechCrops/). Bats have a lower pest-control impact on Bt than on conventional cotton [13]; however, over the past two years, mounting evidence from around the world suggests that insect pests are evolving resistance to Bt-modified crops [18]. While not yet widespread, Bt-resistant pests have been found in the field in India, China, and the U.S., and in laboratory studies [19]–[23].

Here we investigate the impacts of major global market factors – changes in cotton commodity price, the consequent change in the number of hectares planted with cotton, and the adoption of Bt cotton – on the value of the pest-control services provided by Mexican free-tailed bats in the U.S. We calculate service values each year from 1990 through 2008 by estimating both the value of avoided crop damage and the reduced social and private costs of insecticide use in the presence of bats. We assess pest-control services across all U.S. cotton-producing areas containing major Mexican free-tailed bat roosts. Due to the lack of time-series data on the size of bat populations, we could not consider the impact of fluctuations in population size on the value of bat pest control services; as an alternative we analyzed the sensitivity of pest-control values to changes in bat population size. Currently, there is no market-based approach linking bat pest control services to cotton production, just estimates of value that might allow for the development of such mechanisms. As such, we conclude by addressing implications of our valuation research for the development of incentive-based approaches to the conservation of Mexican free-tailed bats in the context of the anticipated increase in Bt-resistant pests and the future value of bat ecosystem services.

## Results

**Figure 1:**
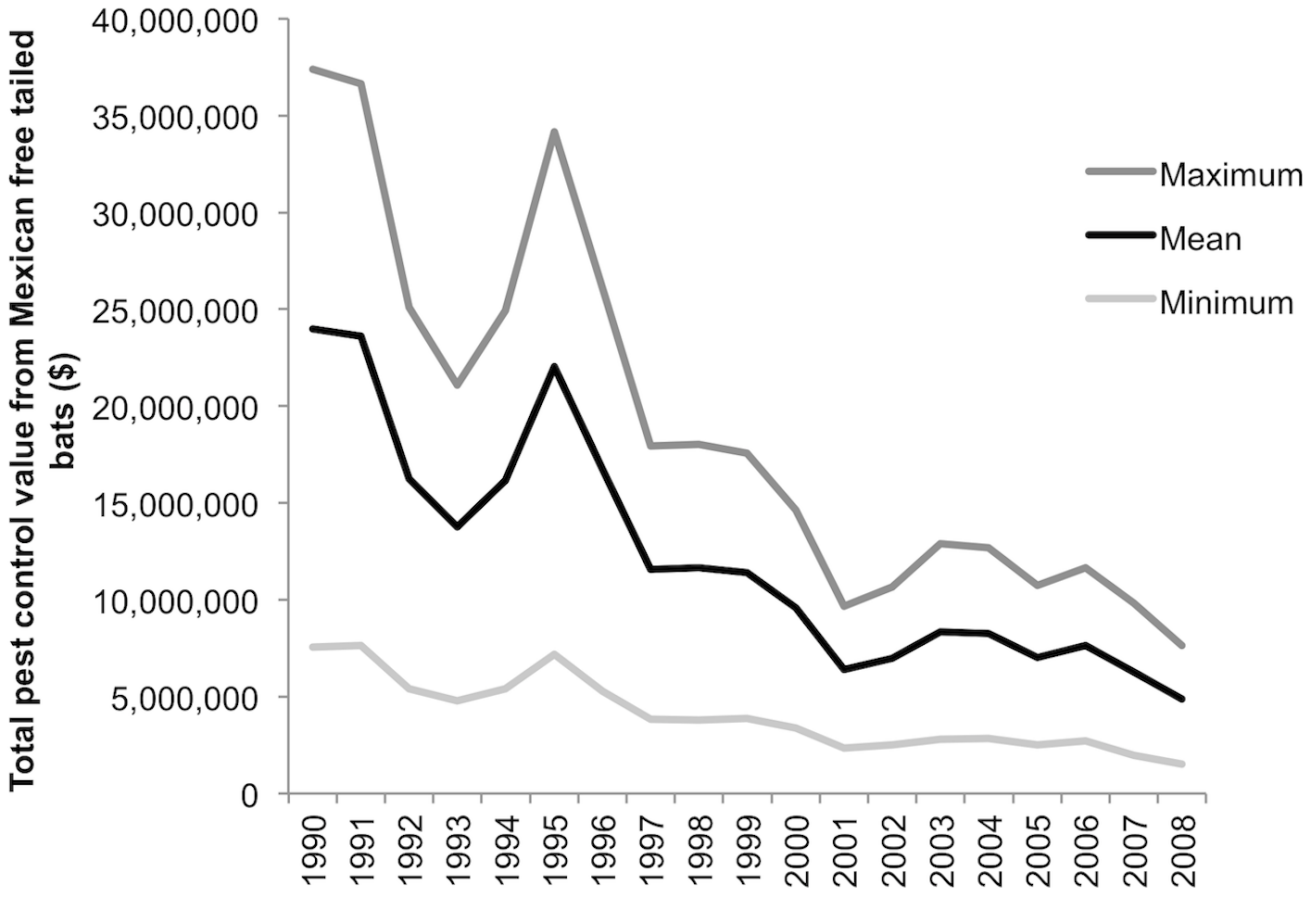
Cotton pest-control value provided by Mexican free-tailed bats over time. Maximum and minimum ecosystem service values for pest control represent calculations using the highest and lowest values, respectively, for several model parameters. From 1990 through 2008, the value of cotton pest-control services across the southwestern U.S. declined by 79%, from a high of $23.96 million in 1990 to a low of $4.88 million in 2008 (mean values). Values are indexed to 2011 U.S. dollars.

From 1990 through 2008, the ecosystem service value of cotton pest-control services provided by Mexican free-tailed bats across the southwestern U.S. declined by 79%, from a high of $23.96 million in 1990 to a low of $4.88 million in 2008 (Fig. 1; mean values; values in 2011 USD). The value notably spiked in 1995 (Fig. 1) due to high prices for Pima and Upland cotton and the high number of hectares planted with cotton in that year (only 1991 had more cotton hectares). The mean annual value of pest control by bats over this time period was $12.24 million (s.d.  =  $6.04 million). The value of pest-control services expressed as a proportion of the total cotton crop value also varied over time, from 28% in 1991 to 6.5% in 2007. The presence of bats in cotton fields precluded, on average, the use of 32,046 kg of insecticide per year and damage to 131,385 kg of cotton per year.

**Figure 2:**
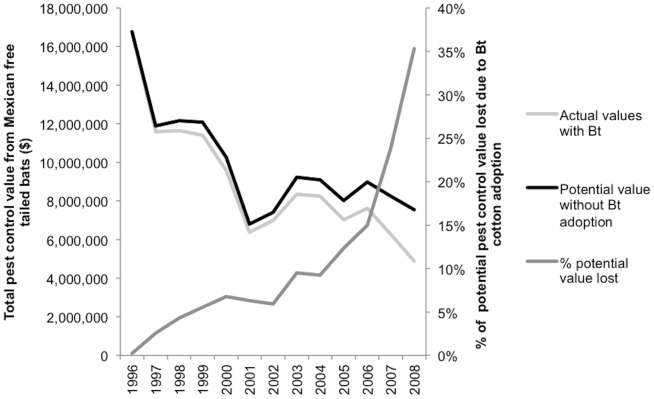
Decreased pest-control value of the bats resulting from adoption of Bt cotton. The light gray line shows actual pest control values from 1996 through 2008. The black line shows the potential value of bat pest control services if Bt had not been adopted. The dark gray line shows the percentage of the potential pest control value lost due to the adoption of Bt cotton. In 2008 the value of bat pest control services was $2.66 million dollars (approximately 33%) less than what it might have been.

Since Bt cotton produces its own insecticide, bats have less of an impact in controlling pests in Bt cotton than they do in conventional cotton [13]. To illustrate the decreased pest-control value of the bats resulting from the adoption of Bt cotton, we used the mean pest-control values (Fig. 1) to calculate what the pest-control values would have been had Bt not been adopted in 1996 (Fig. 2). To do this, we assumed that conventional cotton (i.e. non-Bt cotton) was planted from 1996 to 2008 (Fig. 2). In 2008, for example, the value of bat pest-control services was $2.66 million dollars (approximately 33%) less than what it might have been had all fields been planted with conventional cotton (Fig. 2). Our calculations also indicate that on a per-hectare basis the number of cotton bolls saved by the use of Bt technology was equivalent on average to the foraging efforts of about 27.5 bats.

To better understand temporal variability in ecosystem service values, we assessed the sensitivity of the annualized pest-control value to three factors: total area planted with cotton, the price of cotton, and the Mexican free-tailed bat population size. Notably, the mean pest-control values were equally sensitive to ecological and economic factors: both a ±10% change in the bat population size and a ±10% change in cotton prices caused a ±9.1% change in the mean pest-control values. However, altering the total area planted with cotton by ±10% only caused a ±0.9% change in the mean pest-control value over time.

## Discussion

The results of this study document that volatile market conditions and technological substitutes can affect the value of an ecosystem service [6]. The value of the pest-control services provided by Mexican free-tailed bats in the U.S. declined by 79% from 1990 through 2008 in response to declining global cotton prices, the consequent reduction in the number of hectares planted with cotton, and the introduction and widespread adoption of Bt cotton, a technological substitute to the natural pest-control services of the bats. Our analysis further indicates that these types of changes in market conditions may have as much impact on the value of ecosystem services as changes in ecosystem function such as changes in bat population numbers.

Critics of the ecosystem service approach to biodiversity conservation contend that the risk of diminished service values – like the trends we document here – are a fundamental weakness to “economic benefits” arguments for conservation [3], [4]. The fact that falling cotton prices and the adoption of Bt cotton caused the value of bat cotton pest-control services to fall appears to confirm this concern. However, as the pest-control service of bats depreciates with investments in technology, so might the depreciation of manufactured capital. A recent meta-analysis of studies from five continents indicates that five of thirteen major insect crop pests have evolved Bt resistance in the last eight years, including cotton bollworms [18]. Resistance to Bt can arise in as few as two years depending on local conditions and how carefully growers hew to guidelines for maintaining refuges of non-Bt host plants [18], [24]. Further, the efficacy of the second-generation Bt cotton (called pyramids because they produce more than one type of toxin), can be compromised if local pests are resistant to just one of the toxins [18]. This evidence of resistance evolution suggests that Bt may not be a permanent or even long-term solution to pest-related losses in the production of cotton and other crops, and that bats may again play a critical role in pest control. In fact, by preying on the individual insects that survive the Bt toxin – and preventing them from multiplying – bats may provide the additional service of slowing the evolution of resistance to Bt and other insecticides [13]. Indeed, bats and other natural enemies can play an important role in integrated pest management [25]. For example, Hagerty and colleagues [25] showed that Bollgard II cotton, a two-gene Bt product, experienced increased crop damage when natural predators were disrupted. Many agronomic researchers recommend that Bt crops be used in conjunction with other tactics, including natural predators, to avoid pest outbreaks and to delay the evolution of pest resistance [18], [25].

### The option value of bats: an argument for conservation?

Option values in environmental economics can take one of two forms. The first is a user's willingness to pay for protecting a resource that is not currently in use so that it might be available for future use [26]. The exact value is influenced by the likelihood of needing the resource and the cost of replacing it should it be lost [27], [28]. Alternatively, an option value may be the premium a decision-maker or society is willing to incur to avoid an irreversible loss of a resource by preserving it for future use [29]. In this case, it is possible that the pest-control services of Mexican free-tailed bats will become more valuable again, given that technological replacements for the service run the risk of being temporary. While more work is needed to assess the option value of protecting bats, the notion might provide an intriguing rationale to add to the existing list of reasons for enhancing bat conservation. Conservationists could argue that an investment in protecting bat populations now is an investment in protecting a future stream of potential pest-control services. They might also argue that bats provide pest control free of charge whereas pesticides and Bt cotton are costly to purchase, and their use carries a number of private and social costs. A final point might be that because bats are generalist predators, they provide a broad-spectrum pest control.

This line of reasoning suggests that it might be economically rational for decision-makers to promote bat conservation. However, there is often a disconnection between what is economically profitable and ecologically rational and what is implemented as public policy. Considerable investments have been made in developing Bt and similar pest-control methods and it is likely that such investments will continue [30]. However, we believe that bat conservation is not an alternative, but a rational strategy to use in concert with Bt technology given that neither is completely effective and generalist predators, such as bats, provide broad spectrum pest control and may help slow the evolution of resistance in pest species.

### Have we missed the full value of the ecosystem services provided by Mexican-free-tailed bats?

Critics have long pointed out that it is impossible to identify and measure, let alone value, all the ways functioning ecosystems provide benefits to society [31]–[33]. This leads us to an important caveat to this study – here we have assessed the impacts of one of 44 insectivorous bat species in the U.S. [34] on a single, albeit major pest (*H. zea*) of only one important crop (cotton). Mexican free-tailed bats are generalist predators and can switch diet preferences very quickly [35], [36]. When cotton prices fell and farmers planted other crops instead, the bats likely provided pest-control services to those other crops – indicating that we have not fully accounted for the total value of bats' pest-control services. Finally, a full accounting would consider the other types of services, such as ecotourism, provided by Mexican free-tailed bats [37].

## Conclusion

Valuation of ecosystem services has improved greatly in the last decade [38]–[41]. There is now a rich and growing literature showing how temporal and spatial variation in ecological functions can cause variation in the economic benefits provided by nature [42]–[46]. Our contribution is to demonstrate empirically how fluctuations in market conditions also cause temporal variation in ecosystem service values even when ecosystem function is held constant. Just as values from a particular region should not be blindly transferred to other regions [9], [31], [47], [48], our study illustrates that ecosystem service valuations must also consider how changing market conditions and technological substitutes can alter service values over time. At a minimum, results like ours can be used to develop transfer functions for valuing agricultural pest-control services by accounting for the role of changing agricultural prices and practices in the value of these services [49], [50].

We also hope that these and similar results will spur discussion about the role of ecosystem services in biodiversity conservation in general, and bat conservation in particular. While the value of bat pest-control services to cotton production in the U.S. did indeed decline in response to global market forces and advances in bio-technology, there is a possibility that as pest resistance to Bt cotton rises, bat pest-control service values will rise again. Although currently there are no market-based approaches linking bat pest control services to cotton production, we wonder if the hope of protecting bats now to preserve the “option value” of future services is a sufficient argument to develop incentive-based approaches to bat conservation? Or, is pinning conservation hopes on the notion of option values risky, since we have already witnessed one cycle of technological capital supplanting natural capital? These are the questions we are left to ponder.

## Materials and Methods

### Pest-control value

We employ the avoided-cost approach used by Cleveland et al. [12] to estimate the value of bat services in reducing crop damage and pesticide use on conventional cotton in an eight-county region of South Texas located west of San Antonio. We expand on the approach of Cleveland et al. [12] by considering the effect of the adoption of transgenic Bt cotton in 1996 on bat service values. Our analysis covers the two decades from 1990 through 2008, allowing us to understand how ecosystem service values vary over time as a function of changes in land-use practices and socio-economic factors. In addition, we estimated bat service values across the southwestern U.S., rather than just a region of Texas. This increased geographic scope includes all cotton-producing areas near major Mexican free-tailed bat roosts in the U.S.

Estimating the avoided costs of crop damage involves the following steps: (a) estimating the number of insects consumed nightly by individual bats; (b) determining the hectares of cotton fields within proximity to bat roosts, which allows us to estimate the number of insects consumed nightly by bats in the area of the fields; and (c) the value of the crops that would have been damaged in the absence of bats. To determine the value of reducing insecticide use, we calculated both the reduced private costs to farmers of applying insecticides, and the reduced cost to society of releasing fewer insecticides into the environment. We modified the method of Cleveland et al. [12] to consider the social costs of only those insecticides that specifically target cotton bollworms (*Helicoverpa zea*) [51].

### Bat population estimates and roost locations

Our study area includes all U.S. counties that produce Pima or Upland cotton and that are located within 50 km (conservatively, the bats' nightly foraging distance [52]) of a major Mexican free-tailed bat roost. We obtained data about roosts (location and bat population censuses) from the U.S. Geological Survey's Bat Population Database [53] and our own literature search (Table S1 in File S1). We only considered large summer roosts (>7,000 individuals) because many smaller roosts lack good geospatial information, and because the combined populations of the largest summer colonies are thought to account for the majority (>99%) of the migratory Mexican free-tailed bat population [54]. These major roosts thus provide a reasonable estimate of the number of bats engaged in pest-control services in the U.S. We used only estimates obtained after 1970 to account for concerns that bat populations may have declined in the 1950s and 1960s due to DDT exposure [55], [56]. We assumed that 90% of the adult bats in each colony were female and 10% were male, which is consistent with field data [13]. We did not model changes in the bat population size over time, as the data do not permit time-series analysis [13], but we did analyze the sensitivity of ecosystem service values to a 10% change in bat population numbers (see “Sensitivity analysis of pest-control values”).

### Avoided crop damage calculation

#### Number of pests consumed

We first estimated the value of the crops that would have been lost in the absence of bats providing pest-control services. Conventional and molecular analyses show that moths comprise 30–60% of the bats' diet and indicate that each reproductively active female bat consumes 5–10 female adult bollworms (*Helicoverpa zea*) per night during periods of peak bollworm infestation [12], [35], [36]. Since bollworms also infest other crops in the area or migrate out of the region, we estimated that only 10–20% of the female moths consumed each night (approximately 1.5 individuals per bat) would have dispersed into cotton and laid eggs [12]. Due to high mortality rates during insect development (95–98%), the nightly consumption of 1.5 adult female moths would prevent 5 larvae from developing and damaging cotton crops [12], [57]. Bollworm consumption by non-reproductive females and male bats was calculated as 32% lower than reproductive females due to the high metabolic costs of lactation [12], [13].

For those bollworm that survive development to the larval stage, a single larva can damage 2–3 bolls of cotton over its lifetime [12]. However, because the value of the cotton bolls declines over the season – bolls produced during the first third of season generate about 50% of the harvest while bolls from the last third generate only 7% [58] – we estimated values separately for each third of the season. Further, because bats prevent damage to fewer bolls in Bt versus conventional cotton, we assumed that bats prevented approximately half (52.6%) the number of larvae from developing in Bt versus conventional cotton [13].

#### Cotton locations

We used data from the U.S. Department of Agriculture's National Agricultural Statistics Service (www.usda.nass.gov) and the National Cotton Council (www.cotton.org) on number of cotton hectares planted per county (Table S2 in File S1). Data on numbers of hectares planted with cotton are at the county level, so we approximated locations of cotton fields using crop potential soil maps for each county. This approach assumes that cotton hectares are uniformly distributed over soils with high cotton potential. We used the U.S. General Soil Maps (STATSGO data) from the USDA Natural Resource Conservation Service (NRCS) for locations of soil types suitable for cotton production. For each year from 1990 through 2008, we assumed that the proportion of the cotton hectares planted per county within foraging distance of the bats was equal to the proportion of suitable cotton-growing soils for each county within their foraging range of 50 km from each roost. We also expected that bats disperse randomly from their roost, such that the percentage of the roost's bat population foraging in each cotton-growing area was equal to the percentage of the area each cotton-growing region composed of a roost's total foraging range. Because bats likely disperse non-randomly from their roosts and concentrate on high quality foraging grounds, our calculation is conservative.

### Cotton prices

We used data on cotton prices from 1990 through 2008 from the National Cotton Council. The prices were adjusted for inflation and reported in 2011 USD (SI Appendix, Table S2).

### Avoided insecticide costs calculation

Private costs savings for insecticides reflect the reduced cost to farmers of purchasing and applying chemicals. Data on costs of cotton insecticide applications from 1990 through 2008 were obtained from the Mississippi State University Department of Entomology and Plant Pathology's databases on cotton losses due to insects (http://www.entomology.msstate.edu/resources/cottoncrop.asp). Social cost savings arise from lowered public health impacts to the farm workers who apply the pesticides, and reduced environmental damage due to loss of beneficial pollinators and groundwater contamination [59]. We ascertained the insecticides in the U.S. that are used predominantly on cotton bollworms [60], and used data from Kovach et al. [61] and from Cornell University's Integrated Pest Management Program [62] to estimate the environmental and toxicological impacts of particular cotton insecticides. We then used a pesticide environmental accounting tool [51] to assign a social-cost value in dollars for each insecticide according to the degree of impact estimates. The pesticide accounting tool calculates detrimental impacts in six categories: human health, ground water contamination, aquatic systems (fish), birds, bees, and other beneficial insects. We used a weighted mean cost of insecticide applications per hectare over time.

### Numbers of insecticide applications avoided

Insecticides are generally applied to cotton fields when bollworm infestations reach a threshold of 20,000–25,000 larvae per hectare. The date at which the threshold is reached, which triggers the first insecticide application, varies by region. Regional estimates of dates of first insecticide application were provided by the following cotton pest experts: C Sansone (Texas, Oklahoma, and Kansas), D Munier (California), J Pierce (New Mexico), and P Ellsworth (Arizona). For fields planted with Bt cotton, the threshold is reached later because the bollworm population growth rate is <10% of that in conventional cotton [63], resulting in a lower number of avoided insecticide applications in Bt cotton. We estimated the number of insecticide applications that were avoided in the bats' presence by calculating the number of times the threshold would have been reached without bat predation from the first date of cotton flowering (and susceptibility to bollworms) to the first date of insecticide application. We used a uniform insecticide application rate of 0.29 kg/ha [60]. Finally, we estimated the value of these avoided applications by summing the private and social costs [12], [61]. Data on the cotton season (e.g., mean planting and harvest dates) for different regions were obtained from the USDA's National Agricultural Statistics Service.

### Sources of uncertainty in pest-control estimates

We arrived at high and low estimates of total pest-control services provided by bats using ranges of several parameters for which we did not have accurate estimates. We used the following at their maximum and minimum value: the insecticide application threshold (20,000–25,000 larvae/ha), the number of bolls consumed by a larva over its lifetime (2–3 bolls/larva), and the number of adult female moths dispersing into cotton (0.5–2 individual per bat per night).

### Sensitivity analysis of pest-control values

To better understand factors influencing the ecosystem service values over time, we analyzed the sensitivity of the annualized mean pest-control value over our study period (1990–2008). We altered the following parameters by ±10%: total area planted with cotton, Mexican free-tailed bat population size, and price of cotton. We measured the effect of the parameter alterations on the annualized mean pest control value.

### Impact of Bt on value of pest-control services

Bt cotton was introduced for the Upland variety of cotton in 1996, but is not available for the Pima variety, which accounts for less than 5% of cotton production in the U.S. [64]. Information on the timing of adoption of transgenic Bt Upland cotton was obtained from the Mississippi State University Department of Entomology and Plant Pathology's database on cotton crop losses [64]. To better understand the influence of the adoption of Bt cotton on the bat's pest-control service value, we used the mean pest-control values to calculate potential pest-control values had Bt not been adopted in 1996. To do this, we assumed that conventional cotton (i.e. non-Bt cotton) was planted from 1996 through 2008 and recalculated the pest-control values.

## Supporting Information

File S1 **Contains the following: Table S1 Cotton extent and Mexican free-tailed bat population size per county from 1990 through 2008. Table S2 Upland and Pima cotton price over time.**
(DOCX)
