# Supplementary material for: Market Forces and Technological Substitutes Cause Fluctuations in the Value of Bat Pest-Control Services for Cotton
Source: PLoS One. 2014 Feb 3;9(2):e87912. doi: 10.1371/journal.pone.0087912 (PMC3912186; doi:10.1371/journal.pone.0087912)
Supplement: File S1 — Contains the following: Table S1 Cotton extent and Mexican free-tailed bat population size per county from 1990 through 2008. Table S2 Upland and Pima cotton price over time. (DOCX) [file pone.0087912.s001.docx]

**File S1**

**Table S1. Cotton extent and Mexican free-tailed bat population size per county.**

| **County** | **State** | **Bat population size** | **Mean cotton hectares*** | **County** | **State** | **Bat population size** | **Mean cotton hectares*** |
| --- | --- | --- | --- | --- | --- | --- | --- |
| Pima | AZ | 1,012 | 4,731 | Denton | TX | 24,117 | 641 |
| Colusa | CA | 115 | 1,632 | Donley | TX | 19,754 | 5,677 |
| Sutter | CA | 19,471 | 223 | Floyd | TX | 69,445 | 63,771 |
| Yolo | CA | 50,310 | 421 | Fort Bend | TX | 34,092 | 17,666 |
| Barber | KS | 2 | 97 | Guadalupe | TX | 758,067 | 228 |
| Eddy | NM | 9,263 | 2,897 | Hall | TX | 120,955 | 28,777 |
| Beckham | OK | 91,594 | 4,063 | Hardeman | TX | 2,314 | 4,236 |
| Greer | OK | 133,153 | 3,233 | Harris | TX | 167,276 | 243 |
| Harmon | OK | 114,044 | 8,877 | Hays | TX | 356,558 | 2,119 |
| Harper | OK | 1,553 | 37 | Hidalgo | TX | 362,081 | 27,265 |
| Jackson | OK | 47,544 | 22,425 | Kinney | TX | 59,430 | 2,353 |
| Kiowa | OK | 1,153 | 7,652 | Mason | TX | 353,907 | 30 |
| Pawnee | OK | 18,103 | 175 | McCulloch | TX | 186,304 | 1,937 |
| Payne | OK | 373 | 326 | Medina | TX | 415,902 | 2,391 |
| Woods | OK | 36,261 | 2,700 | Montague | TX | 225,287 | 831 |
| Armstrong | TX | 13,955 | 285 | Motley | TX | 64,320 | 10,526 |
| Bastrop | TX | 203,968 | 399 | Starr | TX | 3,727 | 2,879 |
| Bexar | TX | 1,705,727 | 228 | Swisher | TX | 13,003 | 26,706 |
| Brazoria | TX | 22,780 | 2,195 | Travis | TX | 662,059 | 2,147 |
| Briscoe | TX | 184,253 | 13,636 | Uvalde | TX | 1,063,973 | 3,204 |
| Caldwell | TX | 95,654 | 1,832 | Wheeler | TX | 1,490 | 2,866 |
| Cameron | TX | 2,615 | 26,441 | Williamson | TX | 223,004 | 13,165 |
| Childress | TX | 15,260 | 13,631 | Wilson | TX | 245,298 | 426 |
| Clay | TX | 43,359 | 603 | Wise | TX | 148,727 | 318 |
| Collingsworth | TX | 64,899 | 16,208 | Zavala | TX | 28,255 | 4,127 |

***From 1990 to 2008**

**Table S2. Upland and Pima cotton price over time.**

| **Year** | **Upland Cotton $/lb** | **Pima Cotton $/lb** |
| --- | --- | --- |
| 1990 | 0.75 | 1.12 |
| 1991 | 0.57 | 0.98 |
| 1992 | 0.54 | 0.82 |
| 1993 | 0.66 | 0.88 |
| 1994 | 0.88 | 1.11 |
| 1995 | 0.83 | 1.50 |
| 1996 | 0.71 | 1.08 |
| 1997 | 0.68 | 1.00 |
| 1998 | 0.60 | 0.91 |
| 1999 | 0.52 | 0.81 |
| 2000 | 0.52 | 0.86 |
| 2001 | 0.33 | 0.79 |
| 2002 | 0.47 | 0.80 |
| 2003 | 0.60 | 0.96 |
| 2004 | 0.46 | 1.12 |
| 2005 | 0.49 | 1.13 |
| 2006 | 0.49 | 0.92 |
| 2007 | 0.61 | 0.83 |
| 2008 | 0.48 | 0.94 |
